# Spatial-temporal distribution of dengue and climate characteristics for two clusters in Sri Lanka from 2012 to 2016

**DOI:** 10.1038/s41598-017-13163-z

**Published:** 2017-10-10

**Authors:** Wei Sun, Ling Xue, Xiaoxue Xie

**Affiliations:** 1 Harbin Engineering University, Department of Mathematics, Harbin, 150001 China; 2 University of Manitoba, Department of Mathematics, Winnipeg, R3T 2M8 Canada

## Abstract

Dengue is a vector-borne disease causing high morbidity and mortality in tropical and subtropical countries. Urbanization, globalization, and lack of effective mosquito control have lead to dramatically increased frequency and magnitude of dengue epidemic in the past 40 years. The virus and the mosquito vectors keep expanding geographically in the tropical regions of the world. Using the hot spot analysis and the spatial-temporal clustering method, we investigated the spatial-temporal distribution of dengue in Sri Lanka from 2012 to 2016 to identify spatial-temporal clusters and elucidate the association of climatic factors with dengue incidence. We detected two important spatial-temporal clusters in Sri Lanka. Dengue incidences were predicted by combining historical dengue incidence data with climate data, and hot and cold spots were forecasted using the predicted dengue incidences to identify areas at high risks. Targeting the hot spots during outbreaks instead of all the regions can save resources and time for public health authorities. Our study helps better understand how climatic factors impact spatial and temporal spread of dengue virus. Hot spot prediction helps public health authorities forecast future high risk areas and direct control measures to minimize cost on health, time, and economy.

## Introduction

Dengue (DEN) is a vector-borne disease in humans as a major public health concern in many tropical and subtropical countries^1^. It is the most rapidly spreading mosquito-borne viral disease that has increased 30-fold over the last 50 years, and keeps expanding its geographic distribution globally^2^. The causative agent dengue virus (DENV) consisted of four serotypes is an arbovirus transmitted to humans via the bites of infected *Aedes* mosquitoes. A large proportion of DENV infections, especially in children, progresses from mild dengue fever (DF) to a more severe and life-threatening disease known as dengue hemorrhagic fever (DHF)^3^. The incidences of DF and DHF and the rate of dengue hospitalizations have increased dramatically with the increase of the size, duration, and frequency of dengue epidemics in the past few decades^4,5^, especially in Southeast Asia^6^. The number of dengue cases reported to World Health Organization (WHO) has increased from 0.4 million in 1996 to 3.2 million in 2015^2,7^. It is now estimated that $50{\rm{ \% }}$ of the world’s population are at risk of being infected by DENV^7^. The disease is estimated to place a heavy socio-economic burden on households, health care systems, and governments during outbreaks, particularly for countries with developing economies^8^. Only fluid management and detection of early warning signs of severe disease can be fulfilled by clinical management, while no specific antiviral treatments are currently available. Although the first dengue vaccine, Dengvaxia (CYD-TDV), was registered in Mexico in December, 2015^9^, it is not effective for all strains. Hence, the vaccine is only recommended in regions where there is a high burden of the disease.

Transmission of DENV is mainly through the bites of infected female mosquitoes: *Aedes Aegypti* (*Ae*. *aegypti*) and *Aedes Albopictus* (*Ae*. *albopictus*). *Ae*. *aegypti* is the primary dengue vector in most endemic countries and mainly in urban areas, while *Ae*. *albopictus* serves as a vector primarily in rural areas^10^. The vectors can be transported from regions to regions by wind, vehicles, and airplanes, thus the disease can be transmitted from one location to another. Urbanization, globalization, environment, human behavior, and lack of control of vectors have contributed to the global distribution of DENV^11^. The population dynamics of the vectors is sensitive to environmental conditions^12^, such as humidity, precipitation, and temperature^13–15^.

Sri Lanka has a history of over 40 years of DENV infection with a number of large epidemics in the past decade primarily along the heavily urbanized western coast, and often caused childhood fever. For example, Sri Lanka reported an infection rate of $220$ per $100,000$ people, and approximately a quarter of notified cases occurred in children under $15$ years old living in dengue transmission areas^16^. And since the early 2000s, progressively larger epidemics in Sri Lanka have occurred every regular period of time and increased in magnitude and frequency^17^. In urban areas, the disease incidence is the highest, especially in the district of Colombo, the most densely populated part of the country^18^.

Besides Sri Lanka, dengue remains a major and growing public health problem in many other tropical and subtropical regions or countries, including Brail, and several regions in China etc. Comprehensive understanding of the temporal and spatial patterns of dengue can help develop mitigation strategies to prevent diseases, and save lives and economic costs for countries at risk of dengue transmission. The weekly dengue incidence data for Sri Lanka was made legally reportable since $1996$ ^19^, while the data for many other countries are still not publicly available, too aggregated, or missing. The analysis of the relationships between climatic factors and dengue epidemics in Sri Lanka may help understand the impact of climatic factors on the emergence and reemergence of dengue, and help design effective mitigation and control strategies for dengue in other tropical or subtropical regions.

Climate plays a major role in global and local spared of dengue as an environmental factor due to the impacts on dengue vector life cycle and the vectors’ ability to spread the disease among humans^20^. The climate of the island could be characterized as tropical due to its geographic location with two monsoons, the northeast monsoon (December to March) and the southwest monsoon (May-September). The two monsoons and the convectional activity during the intervening periods are the main origins of rainfall in Sri Lanka^21^. The average annual temperatures for the country range from $28$ to 30 °C, and humidity ranges from 60 to 90% during different seasons in different regions of the country^22^. The mean annual temperatures are largely homogeneous in the low lands and rapidly decreasing in the highlands^23^. Elevated rainfall affects vector abundance by increasing immature mosquito habitats and stimulating egg hatching^24^. Even though heavy rainfalls may transiently reduce the risk of transmission by flushing larvae and pupae away from breeding sites or killing them, elevated humidity can increase mosquito survival^25^. Hence, the risk of dengue transmission may increase with elevated temperature, rainfall, and humidity by increasing the reproductive rate.

Since the distribution of DENV is spatially heterogeneous, identifying the spatial distribution and spatial clusters of dengue and recognizing the association of dengue incidences with climatic factors may be an efficacious strategy to target surveillance and control efforts in a cost-effective manner, particularly in Sri Lanka where dengue is hyper-endemic and public health resources are scarce. The reported monthly dengue incidence data and daily meteorological data for various districts in Sri Lanka allowed exploring correlation between variation of dengue incidences and meteorological factors. The analysis helps us deduce spatial evolution of dengue epidemic and determine high dengue incidences and clusters of regions with similar climatic characteristics of dengue epidemic.

Spatial-temporal analysis was widely used in detecting distribution patterns of infectious diseases^26^. Hot spot analysis is an important spatial analysis tool to identify unusual aggregations of epidemiological events and predict high risk areas of disease transmission. It is commonly used in detecting distribution patterns of diseases, identifying a locality with active disease transmission, and evaluating the association between disease incidence and climatic factors, ecological, socio-economic, and demographic factors^27,28^. Spatial-temporal clustering analysis is a method for grouping objects based on their similarities in space and time. As a subfield of data mining, this method has gained high popularity in many fields, especially in geographic information science. Hence, spatial hot spot analysis and spatial-temporal clustering analysis are useful for disease surveillance, spatial and temporal epidemiology, population genetics, landscape ecology, crime analysis, and many other fields, and they play an important role in quantifying geographic variation patterns.

Several previous studies also focused on the distribution of dengue incidences by detecting its association with climatic factors for specific study areas. Bostan *et al*. analyzed the impact of temperature and precipitation on dengue epidemic, and found that increased sustenance of dengue infection can be explained by average temperature and precipitation^29^. Sirisena *et al*. showed that dengue incidences have positive correlation with rainfall prior to two to five months, and positive correlation with a rise in temperature prior to nine months, while no positive correlation with temperature or humidity^30^. Sumi *et al*. analyzed climatic data and dengue incidence data for Manila of Philippines using time-series analysis, spectral analysis and the least squared method, and showed that dengue incidences are correlated not only with precipitation, but also relative humidity and temperature^31^. Johansson *et al*. demonstrated that short-term and seasonal autocorrelation were keys to improving short-term and long-term forecasts by analyzing dengue epidemic in Mexico^32^. Ehelepola *et al*. showed that dengue incidences have negative correlation with large diurnal temperature ranges by analyzing the relationship between dengue incidences and temperature^33^. Choi *et al*. analyzed three provinces in Cambodia, and demonstrated that mean temperature and rainfall have significant association with dengue incidences^34^. They also showed that the association between dengue incidences and climatic factors apparently varies by locality, and dengue warning system should be implemented at a local or regional scale^34^. These analysis are helpful to implement effective measures for local governments.

Understanding spatial distribution and climatic characteristics of dengue can be helpful for Sri Lanka to implement controlling measures for different regions. Since the dynamics of mosquitoes and dengue epidemic vary in different regions of Sri Lanka, applying the same control measures for all 25 districts may not be effective. As practical experience showed that controlling dengue should be a regional effort, applying mitigation strategies only in one district may fail if its neighboring districts do nothing in controlling the spread of dengue virus^35^. Therefore, identifying important epidemic areas with similar incidences of dengue and revealing the common climatic characteristics of dengue epidemic for these areas are prerequisites of implementing cooperative measures on controlling the spread of dengue virus effectively.

The aims of this study are to analyze the spatial distribution of dengue, identify important dengue epidemic areas of Sri Lanka, and investigate the association of dengue cases with climatic factors, so as to strengthen the basis of dengue mitigation strategies in this high-burden country. We have evaluated the spatial and temporal distribution of dengue in all the 25 districts, forecasted dengue incidences and hot and cold spots, given real-time meteorological data and historical dengue incidences, and derived the association of geographic and climatic risk factors with dengue incidences.

## Results

### Descriptive analysis

Table 1 lists the number of dengue cases in all the 25 districts of Sri Lanka from 2012 to 2016. The total number of cases over these years is high in areas with large population. Colombo reported the largest total number of dengue cases, accounting for nearly a third of the total number of cases in the whole country from 2012 to 2016. Besides, Colombo district reported the highest dengue caseload for any given year. The population in Colombo is around $2.3$ million, approximately $12{\rm{ \% }}$ of the national population, $20.4$ million. Gampaha had the second largest total number of dengue cases over the years, which is nearly $\mathrm{15.42 \% }$ of the total for the whole nation. Ratnapura had the third largest total number of dengue cases from 2012 to 2016. The population densities in Colombo, Gampaha, and Ratnapura were the largest, second, and third, and the numbers of dengue cases for these three districts were in the same order. The smallest total number of dengue cases occurred in Kilinochchi located in the north of Sri Lanka with low population density. The total number of dengue cases in the whole nation reached the peak in 2016. Large numbers of dengue cases mostly occurred in Colombo and its surrounding areas, while small numbers of cases mostly occurred in the north of Sri Lanka. The number of dengue cases in Jaffna kept increasing from 2012 to 2016.

**Table 1:** The number of dengue cases for each district of Sri Lanka for 2012 to 2016 collected from Epidemiology Unit of Sri Lanka^36^.

District	2012	2013	2014	2015	2016	Total
Ampara	155	253	158	67	260	893
Anuradhapura	493	524	632	401	731	2781
Badulla	430	534	1113	566	1185	3828
Batticaloa	717	518	970	1474	612	4291
Colombo	10017	10489	14711	9881	16767	61865
Galle	1513	915	1224	1030	3086	7768
Gampaha	8006	3525	8811	4142	7173	31657
Hambantota	604	361	665	398	900	2928
Jaffna	894	709	1839	2016	2468	7926
Kalutara	2791	1962	2631	1559	3502	12445
Kandy	2517	1618	2336	1325	4063	11859
Kegalle	2705	1103	1724	711	1513	7756
Kilinochchi	93	85	90	92	86	446
Kurunegala	3537	2227	2464	1253	2556	12037
Mannar	186	58	359	105	232	940
Matale	596	462	649	401	1148	3258
Matara	1835	538	748	459	1384	4964
Moneragala	287	368	313	223	475	1666
Mulativu	42	167	134	142	182	667
Nuwara Eliya	342	312	314	180	421	1569
Polonnaruwa	289	515	558	250	479	2091
Puttalam	1800	829	916	739	1046	5330
Ratnapura	398	1732	2823	1041	3130	12664
Trincomalee	168	902	661	587	503	2821
Vavuniya	104	94	142	197	268	805
Total	44059	30802	46985	29239	54170	205255

Spatial-temporal analysis has been conducted to identify transmission dynamics of dengue from 2012 to 2016 in Sri Lanka. Table 2 showed dengue incidence, defined as the number of cases per $10,000$ population, for all districts from 2012 to 2016. Dengue incidences for Colombo from 2012 to 2016 were all above $40$, which were the highest among all the other districts. The dengue incidence in Jaffna, located in the north of Sri Lanka, increased rapidly from $15.28$ to $41.00$, and was much more than the incidence of Gampaha in 2015 and 2016. Most districts in the north had small numbers of dengue cases and low incidences.

**Table 2:** The dengue incidences for each district of Sri Lanka from 2012 to 2016.

District	2012	2013	2014	2015	2016
Ampara	2.38	3.84	2.37	0.10	3.84
Anuradhapura	5.71	6.00	7.16	4.49	8.08
Badulla	5.26	6.46	13.33	6.71	13.88
Batticaloa	13.58	9.76	18.13	27.25	11.20
Colombo	42.99	44.84	62.41	41.60	70.01
Galle	14.19	8.52	11.31	9.44	28.00
Gampaha	34.66	15.17	37.69	17.47	30.24
Hambantota	10.0.03	5.73	10.74	6.34	14.13
Jaffna	15.28	12.04	31.01	33.77	41.00
Kalutara	22.78	15.91	21.20	12.47	27.77
Kandy	18.23	11.63	16.66	9.36	28.33
Kegalle	32.09	12.99	20.16	8.26	17.41
Kilinochchi	8.16	7.33	7.63	7.67	6.99
Kurunegala	21.78	13.63	14.98	7.56	15.25
Mannar	18.60	5.74	34.85	10.10	21.89
Matale	12.26	9.41	13.08	7.99	22.60
Matara	22.46	6.53	9.00	5.48	16.38
Moneragala	6.34	8	6.72	4.72	9.92
Mulativu	4.52	17.96	14.26	15.11	19.16
Nuwara Eliya	4.78	4.31	4.28	2.43	5.63
Polonnaruwa	7.08	12.44	13.45	5.97	11.27
Puttalam	23.53	10.75	11.74	9.35	13.06
Ratnapura	3.64	15.72	25.32	9.24	27.46
Trincomalee	4.41	23.43	16.91	14.79	12.45
Vavuniya	6.01	5.37	8.02	11.01	14.73

Dengue incidence is the number of cases per 10000 population. The population data for each district of Sri Lanka were collected from Department of Census and Statistics^37^.

### Prediction with historical dengue incidence data

Association of dengue incidences with local climatic factors was evaluated by the Spearman’s correlation test at $0.05$ significance for all $25$ districts. The results showed that there were certain correlations between dengue incidences and local climates, and the correlations are different for different districts.

**Figure 1:**
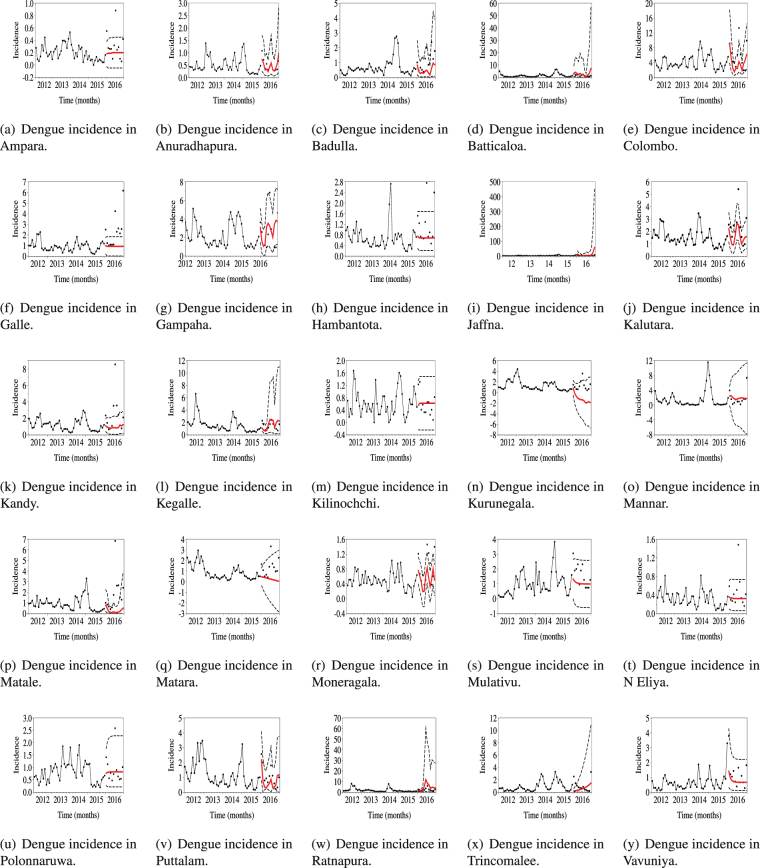
Annual dengue incidences for 25 districts of Sri Lanka from 2012 to 2016. We predicted dengue incidences for 2016 with dengue incidence data from 2012 to 2015 and meteorological data for 2016. The $\bullet$ represents incidence data, the red lines represent the predicted data, and the dotted lines represent the confidence interval.

Next, ARIMAX models were generated for each district to predict future trends of dengue incidences. For each ARIMAX model, the dependent variable was dengue incidence, and the explanatory variable was chosen from local correlated climatic factors for each district. Dengue incidence data for 2012 to 2016 were collected from Epidemiology Unit of Sri Lanka^36^. Using data up to 2015, dengue incidences for 2016 were predicted. As shown in Fig. 1, most of the dengue incidences of 2016 fell into the forecasted regions.

### Spatial distribution with hot and cold spots analysis

A hot spot is defined as an area with great transmission within its neighborhoods. On the contrary, a cold spot is an area with low transmission among nearby locations. Getis-Ord Gi* statistic was used to detect hot spots of dengue epidemic in Sri Lanka for each year from 2012 to 2016. High GiZScores are associated with hot spots, and low GiZScores are associated with cold spots. Table 3 showed that hot spots, in 2012–2016, were mainly located in the west coast of Sri Lanka near Colombo. The hot spots tended to shift eastward to the east coast of Sri Lanka, and northward to Jaffna. Table 3 showed that cold spots of dengue incidences were mainly located in two areas: the southern area surrounding the Moneragala district and the mid-northern area adjacent to the Anuradhapura district. Thus, the districts in the south and mid-north of Sri Lanka were at low risk.

**Table 3:** Results of hot spots analysis on dengue incidences, Sri Lanka in 2012–2016.

GiZScores	2012	2013	2014	2015	2016	Prediction for 2016
>1.50 Standard Deviation	Colombo Gampaha Ratnapura	Colombo	Colombo	Colombo Jaffna	Colombo	Colombo Jaffna
0.50~1.50 Standard Deviation	Kalutara	Badulla	Badulla	Ampara	Badulla	Ampara
	Kegalle	Kalutara	Gampaha	Badulla	Galle	Batticaloa
	Kurunegala	Kurunegala	Jaffna	Batticaloa	Gampaha	Gampaha
	Matara		Mannar	Ratnapura	Jaffna	
	Puttalam		Ratnapura		Kalutara	
					Kandy	
					Kegalle	
−0.50 ∼ 0.50 Standard Deviation	Ampara	Ampara	Ampara	Galle	Ampara	Anuradhapura
	Batticaloa	Batticaloa	Batticaloa	Gampaha	Kurunegala	Badulla
	Galle	Galle	Kalutara	Kalutara	Mannar	Galle
	Jaffna	Gampaha	Kandy	Kandy	Matale	Kalutara
	Kandy	Jaffna	Kegalle	Kegalle	Matara	Kandy
	Mannar	Kandy	Kurunegala	Mannar	Mullaitivu	Kegalle
		Kegalle	Mullaitivu	Mullaitivu	Ratnapura	Kurunegala
		Matale	Nuwara Eliya	Puttalam	Trincomalee	Mannar
		Mullaitivu	Polonnaruwa	Trincomalee	Vavuniya	Mullaitivu
		Polonnaruwa	Trincomalee	Vavuniya		Polonnaruwa
		Puttalam				Ratnapura
		Ratnapura				Trincomalee
		Trincomalee				
<−0.50 Standard Deviation	Anuradhapura	Anuradhapura	Anuradhapura	Anuradhapura	Anuradhapura	Kilinochchi
	Badulla	Hambantota	Galle	Hambantota	Batticaloa	Hambantota
	Hambantota	Kilinochchi	Hambantota	Kilinochchi	Hambantota	Matale
	Kilinochch	Mannar	Kilinochchi	Kurunegala	Kilinochchi	Matara
	Matale	Matara	Matale	Matale	Moneragala	Moneragala
	Moneragala	Moneragala	Matara	Matara	Nuwara Eliya	Nuwara Eliya
	Mullaitivu	Nuwara Eliya	Moneragala	Moneragala	Polonnaruwa	Puttalam
	Nuwara Eliya	Vavuniya	Puttalam	Nuwara Eliya	Puttalam	Vavuniya
	Polonnaruwa		Vavuniya	Polonnaruwa		
	Trincomalee					
	Vavuniya					

Hot and cold spots were identified according to dengue incidences and climatic factors in Sri Lanka from 2012 to 2016. *Getis–OrdG*_i_^*^ statistic was used to detect hot spots of dengue epidemic in Sri Lanka from 2012 to 2016. High GiZscores are associated with hot spots, and low GiZscores are associated with cold spots. Hot spots were mainly located in west coast of Sri Lanka near Colombo. The hot spots tended to shift eastward to the east coast of Sri Lanka, and northward to Jaffna. Cold spots were mainly distributed in the southern area surrounding the Moneragala district, and the mid-northern area adjacent to the Anuradhapura district. The last column is prediction for hot spots and cold spots of 2016 with the predicted dengue incidences in Fig. 1 for 2016.

Hot and cold spots predicted using the predicted dengue incidences for 2016 shown in Fig. 1 were listed in the last column of Table 3. Colombo, Gampaha, and Jaffna districts were predicted as hot spots. Puttalam and Moneragala were predicted as cold spots. Most of the hot and cold spots of 2016 in Sri Lanka identified by analysis were included in our prediction.

### Spatial-temporal clustering analysis

During the study period, two spatial-temporal clusters were formed according to the similarity of dengue incidences in space and time. Table 4 indicated that dengue incidences showed significant spatial-temporal associations, and $p$-value $\mathrm{ < 0.001}$, and two Log likelihood ratios were all greater than the critical value, $12.36$, for the standard Monte Carlo at $\mathrm{5 \% }$ significance. The two clusters were all statistically significant. The first cluster only included Colombo in the hot spot, and the clustering period was from May 2014 to September 2016. The second cluster included 14 districts in the cold spots with clustering period from June 2013 to November 2015, and the clustering center was located in (7.94 N, 81.00E) and the radius was 129.69 km.

**Table 4:** The characteristics of each cluster.

Variables	The first cluster	The second cluster
Coordinates/radius	(6.93 N, 79.85 E)/0 km	(7.94 N, 81.00E)/129.69 km
Time frame	from May 2014 to September 2016	from June 2013 to November 2015
Observed/expected	3.10	0.56
Relative risk	3.07	0.50
Log likelihood ratio	17196.38	9887.81
*p*-value	<10^−17^	<10^−17^

The first cluster includes Colombo, and the second cluster includes Ampara, Anuradhapura, Badulla, Batticaloa, Kurunegala, Kandy, Kegalle, Matale, Moneragala, Nuwara Eliya, Polonnarwa, Puttalam, Trincomalee, and Vavuniya.

### Climate characteristics of the spatial-temporal clusters

**Figure 2:**
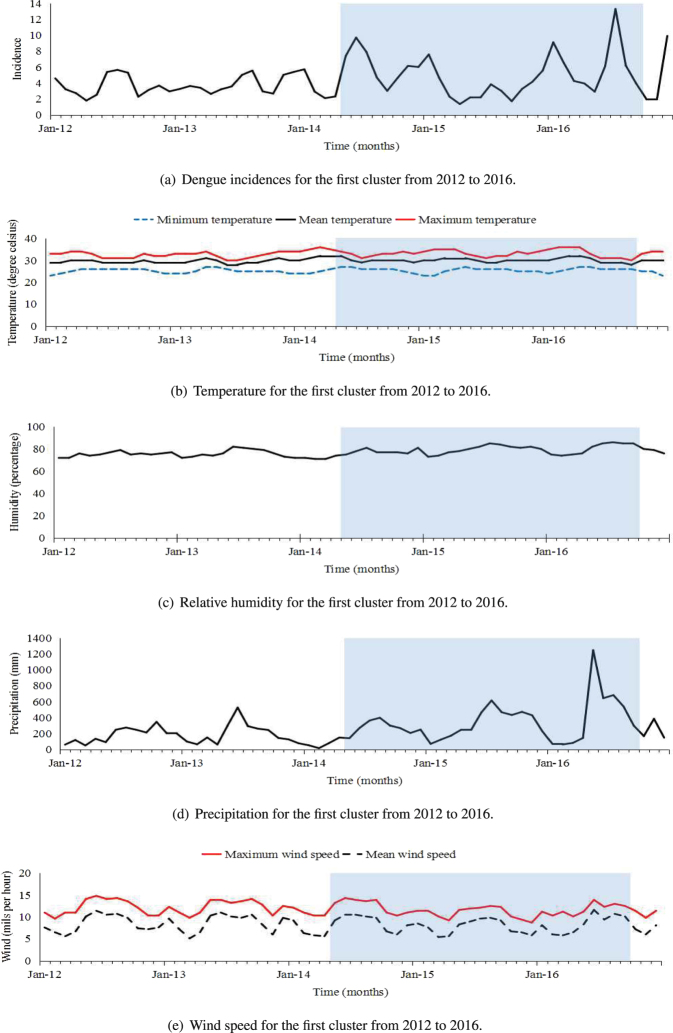
Dengue incidences and monthly climatic factors between 2012 and 2016 for the first cluster. The clustering period is highlighted in blue from May 2014 to September 2016..

**Figure 3:**
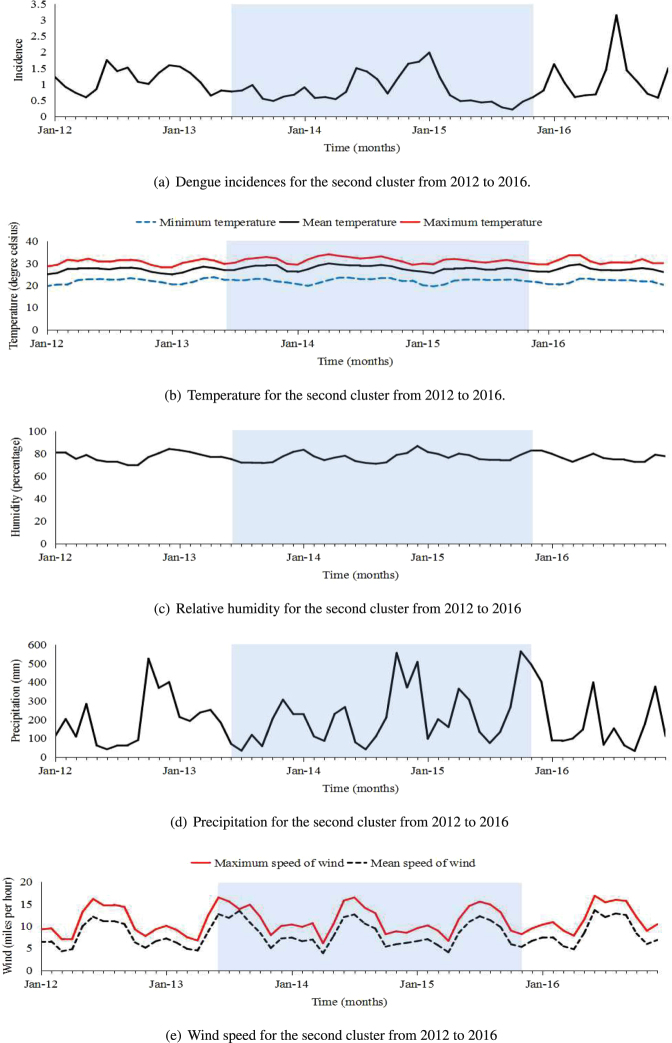
Dengue incidences and monthly climatic factors between 2012 and 2016 for the second cluster. The clustering period is highlighted in blue from June 2013 to November 2015.

For the two special-temporal clusters, Figs 2 and 3 showed the trends of dengue incidences varying with climatic factors. For each cluster, the incidences during clustering period were different from those during non-clustering period.

For the first cluster, Fig. 2 indicated that dengue incidences mainly peaked around July and December annually, and the lowest dengue incidences mainly occurred around April and September annually. The dengue incidences occurred during the clustering period were much higher than the incidences occurred during the non-clustering period. Further, Table 5 showed that during the clustering period, sample means and variances for temperature, humidity, and precipitation were larger than those during non-clustering period, while the sample means and variances for wind speed were smaller than those during non-clustering period.

For the second cluster, Fig. 3 showed that dengue incidences mainly peaked around April and June annually. We found that the dengue incidences occurred in the clustering period were much lower than those occurred during the non-clustering period. Hence, the clustering period, from June 2013 to November 2015, can be considered as a low-incidence period. The sample means and variances of climatic factors for clustering and non-clustering periods were different. During the clustering period, the sample mean of the humidity was smaller, while the sample means of the maximum and mean wind speed were much larger, and the sample means for precipitation and temperature (minimum, average and maximum) were slightly larger than the corresponding sample means during non-clustering period. The sample variances for the minimum and maximum temperature were smaller, and the sample variances for the average wind speed was much larger than the corresponding sample variances during non-clustering period (Table 5).

**Table 5:** Description on the incidences and climatic factors for two clustering areas.

Climate variable	The clustering period			Non-clustering period		
	Mean	Median	Variance	Mean	Median	Variance
Incidence^[*a*]^	4.89	4.24	7.38	3.84	3.23	2.91
Minimum temperature^[*a*]^	25.61	26.00	1.11	25.03	25.00	1.11
Mean temperature^[*a*]^	30.19	30.00	1.16	29.55	30.00	0.61
Maximum temperature^[*a*]^	33.32	33.00	3.03	32.66	33.00	1.81
Humidity^[*a*]^	78.97	78.00	17.57	75.69	76.00	9.08
Precipitation^[*a*]^	327.08	267.61	61159.66	181.71	149.56	13746.66
Maximum wind speed^[*a*]^	11.55	11.40	2.28	12.08	12.10	2.56
Mean wind speed^[*a*]^	8.09	8.30	3.51	8.35	8.10	3.52
Incidence^[*b*]^	0.83	0.66	0.20	1.16	1.07	0.28
Minimum temperature^[*b*]^	22.08	22.4	1.14	21.85	22.1	1.20
Mean temperature^[*b*]^	27.84	27.73	1.30	27.11	27.40	1.23
Maximum temperature^[*b*]^	31.41	31.57	1.76	30.68	30.73	1.82
Humidity^[*b*]^	76.76	76.37	16.91	77.03	77.00	14.81
Precipitation^[*b*]^	220.44	203.28	24058.78	186.47	149.82	17854.98
Maximum wind speed^[*b*]^	11.51	10.56	9.67	11.09	11.09	9.78
Mean wind speed^[*b*]^	8.39	7.57	8.22	7.96	7.04	7.98

The clustering period is from May 2014 to September 2016 for the first clustering area, and is from June 2013 to November 2015 for the second clustering area. The other period denotes the period from 2012 to 2016 except the clustering period. We let ^[a]^denote climate variables for the first clustering area, and let ^[b]^denote variables for the second clustering area.

## Discussion and Conclusion

Dengue is a major public health problem affecting more than half of the world’s population living in tropical and subtropical regions of the world and has become a high priority disease for public health authorities in Sri Lanka since 1960s. Dengue is an all-year-around disease in Sri Lanka with peak number of reported dengue cases usually occurring during the two monsoons. The disease is estimated to place a heavy socio-economic burden on health care systems and governments due to the involvement of large numbers of public health staff in dengue control activities and the provision of free medical care to dengue patients at secondary care hospitals. This study is designed to identify the climatic factors, geographic locations, and population density that are associated with dengue risks to facilitate public health authorities to promptly target the high risk areas.

Spatial analysis with hot spots is suitable to focalize health control measures and epidemiological surveillance in a cost effective manner particularly in regions where resources are limited, like Sri Lanka and many other countries. Moreover, spatial-temporal clustering analysis for detecting important dengue epidemic areas is also of great importance for the prevention and control of dengue and many other vector-borne diseases. We detected the distribution of dengue incidences in Sri Lanka in space and time, important areas of dengue epidemic, and further investigated the climate characters of dengue incidences for these important epidemic areas. One spatial-temporal cluster is the Colombo district and the other cluster includes the southern and mid-northern districts.

The major outbreaks keep appearing in the Colombo district and its surrounding districts. Colombo district is located in the most urbanized Western Province and in the wet zone of the Ceylon island. The weather is wet and tropical, which is favorable for breeding and survival of mosquitoes. Colombo has reported the largest number of cases among all the districts with peaks appearing in July and December, during the two monsoons each year. Colombo has a population of 2.3 million, accounting for $\mathrm{11.5 \% }$ of the total population of the country^37^. Moreover, Colombo is the commercial and administrative hub of the country with around one million people commuting in and out of the city daily^21^. High temperature, high humidity, heavy rainfall, low wind speed, high population density, and large mobility contribute to high dengue incidences in Colombo.The public sector costs of dengue control activities and the direct costs of hospitalizations in Colombo district were estimated to be high in this most heavily populated and urbanized district in Sri Lanka during the epidemic year of 2012 from the Ministry of Healths perspective^8^.

The 14 districts in the second spatial-temporal cluster, which are mainly located in the areas of cold spots from 2012 to 2016, had consistently low dengue incidences between June 2013 and November 2015. These 14 districts are mainly located in the mid-north and south of Sri Lanka. The humidity and wind speed were found to have association with humidity and wind speed. During the clustering period, the sample mean of humidity was lower and the sample mean of wind speed was higher than corresponding ones during non-clustering period.

The analysis showed that climatic characteristics of dengue incidences for these two spatial-temporal clusters are different, then intervention measures on dengue for these regions should focus on different aspects. Firstly, the climate characteristics of dengue incidences in Colombo was obvious, that is, a strong association between the dengue incidences and climatic factors existed. Therefore, prevention and control measures should focus on the higher incidences caused by climate change. Secondly, for the 14-districts in the south and mid-north of Sri Lanka, the association of dengue incidences with climatic factors is weak, then the important driving factors of dengue transmission may be some non-climate factors. Therefore, prevention and control measures on dengue in this region should focus on other non-climate factors, such as environmental health, social media, human behavior change, and immigration of infectious agents etc.

Since daily or weekly dengue incidence data are not publicly available, we only considered relationships between dengue incidences and climatic factors for two important areas, while other areas of Sri Lanka are not discussed specifically. When the daily or weekly dengue incidence data are available, we could explore more on the distribution and climatic characteristics of dengue for non-clustering regions in Sri Lanka.

In this study, the prediction models (ARIMAX) were used to forecast dengue incidences with past incidence data and current meteorological data. The given incidence data points aligned within the forecasted area showed the reliability of these models in predicting future outbreaks. Prediction models can play an important role in controlling and predicting dengue outbreak effectively in Sri Lanka. Further, our findings contribute to a better comprehension of the spatial evolution of dengue by assessing the relationship between disease clusters and climatic factors. These results can assist public health authorities with planning surveillance and control activities according to the weather. Focalizing dengue control measures for disease clusters may significantly reduce virus transmission in comparison with random interventions. Further studies are needed to define whether these hot spots can sustain over time and whether other hot spots arise. Similar studies can be applied to other vector-borne infections, such as Rift valley fever, Chagas, Chikungunya, and Zika virus affecting not only Sri Lanka but also other tropical and subtropical countries.

## Methods

### Study area

Sri Lanka is an island in India Ocean located in the tropic area, spanning approximately $65.61$ thousand square kilometers. This country is classified into nine provinces and 25 administrative districts. According to the latest census in $2012$, Sir Lanka has a population of approximately $20$ million, and the density is $309$ per square kilometer. The density of population varies across the country^38^.

The country is consisted of three agro-climatic zones, wet, dry and inter-mediate zones. In Sri Lanka, climatic characteristics vary for different geographical environment. Generally, its upland areas are cooler, while the coastal areas are warmer. The climate of the island is affected by two seasonal monsoons. One is the Northeast monsoon, which occurs from November to late February annually and affects the whole island. The other is the Southwest monsoon, which occurs in June and July annually and only affects the areas in the central highlands and southwest of Sri Lanka^39^.

### Data Collection

This study is mainly focused on $25$ districts of Sri Lanka. The monthly number of dengue cases for each district between January $2012$ and December $2016$ were obtained from the epidemiology Unit, Ministry of Health, Sri Lanka^36^. The latest available population data were collected from the estimates calculated in 2012 by Department of Census and Statistics^37^. The source and units of dengue disease data, population data, latitude and meteorological data were shown in Table 6. Dengue incidences at district level were calculated by the following formula:

1 $$Incidence=\frac{Number\,of\,cases}{Total\,population\,at\,risk}\times \mathrm{10,000.}$$

**Table 6:** Sources of the data.

Data	Unit	Source
Dengue cases	dimensionless	36
Population	dimensionless	37
Longitude,Latitude	degree	23
Mean temperature	°*C*	23
Maximum temperature	°*C*	23
Minimum temperature	°*C*	23
Relative humidity	°*C*	23
Precipitation	*mm*	23
Mean wind speed	*mm*	23
Maximum wind speed	*mm*	23

Since monthly population data are not available, we used the mid-year population collected from Department of Census & Statistics of Sri Lanka^37^ as an approximation of the monthly data.

### Data Analysis

The ARIMA (AutoRegressive Integrated Moving-Average) model methodology proposed by box and Jenkins analyzes univariate stochastic time series, i. e. error term of this time series^40^. The prerequisite for using this method is that the mean, variance and covariance of the series are all constant over time. All the typical results of the classical regression analysis are not valid for non-stationary series. Empirical studies found that including leading indicators into the model improves forecasting performance. The series ${Y}_{t}$ is said to be $ARIMA(p,d,q)$ if

2 $$\varphi (L\mathrm{)(1}-L{)}^{d}{Y}_{t}=\theta (L){\varepsilon }_{t},$$

where ${\varepsilon }_{t}$ is white noise and no common factors exist between autoregressive polynomial, $\varphi (L)=1-$ ${\varphi }_{1}L-\cdots -{\varphi }_{p}{L}^{p}$, and moving average polynomial, $\theta (L\mathrm{)=1}+{\theta }_{1}L+\cdots +{\theta }_{q}{L}^{q}$, where $L$ is a lag operator. The ARIMA model is extended into ARIMA model with explanatory variable (X), called $ARIMAX(p,d,q)$. Specifically, $ARIMAX(p,d,q)$ can be represented by

3 $$\varphi (L\mathrm{)(1}-L{)}^{d}{Y}_{t}={\rm{\Theta }}(L){X}_{t}+\theta (L){\varepsilon }_{t}\mathrm{.}$$

In this study, correlation analysis and ARIMAX model were used to elucidate the association of climatic factors with dengue incidences and forecast future outbreaks. The analysis was implemented by the SPSS software (version 19). For the independent variable $X$, we selected the climatic factors that have high correlation with dengue incidences. The values of $p$, $d$ and $q$ are determined by the software by minimizing the error between the data and the prediction.

Hot and cold spots at the district level were determined by the hot spot analysis tool built in ArcGIS 10.5^41–43^. Hot spots for dengue incidences were identified by the Getis-Ord ${G}_{i}^{\ast }(d)$ statistic, which is defined as follows^44^:

4 $${G}_{i}^{\ast }(d)=\frac{\sum _{j\mathrm{=1}}^{N}{w}_{ij}(d){x}_{j}}{\sum _{j\mathrm{=1}}^{N}{x}_{j}},$$

where ${w}_{ij}(d)$ is the spatial weight between district $i$ and $j$, and ${x}_{j}$ is the dengue incidence for district $j$, and $N$ is the total number of districts. The ${G}_{i}^{\ast }$ statistic was used to test the statistically significant autocorrelation of dengue cases, and to determine the spatial dependence of nearby observations, for each year. In order to improve the performance of this statistical test, Ord and Getis (1995)^45^ extended this statistic to a standardized form, which is equivalent to the z-value of ${G}_{i}^{\ast }$,

5 $$Z({G}_{i}^{\ast }(d))=\frac{{G}_{i}^{\ast }(d)-E({G}_{i}^{\ast }(d))}{\sqrt{Var({G}_{i}^{\ast }(d))}},$$

where the expectation and variance of ${G}_{i}^{\ast }$ are as follows:

6 $$E[{G}_{i}^{\ast }(d)]=\frac{1}{N}\sum _{j\mathrm{=1}}^{n}{w}_{ij}(d),$$

7 $$Var[{G}_{i}^{\ast }(d)]=\frac{1}{{N}^{2}(N-\mathrm{1)}}{(\frac{s}{\bar{x}})}^{2}(N\sum _{j\mathrm{=1}}^{n}{w}_{ij}^{2}(d)-{(\sum _{j\mathrm{=1}}^{n}{w}_{ij}(d))}^{2})\mathrm{.}$$

Here $s$ is the standard variance and $\bar{x}$ is the sample mean. In this study, the weights are determined by Thiessen polygons based on regions with similar climate and short distances. Thiessen Polygons are Voronoi Cells that divide an area given a set of known values at a relatively small number of points.

Spatial-temporal distribution of dengue in Sri Lanka between 2012 and 2016 was identified by Kulldorff’s spatial scan statistic with searching spatial-temporal cylinders using SaTScan software. The spatial-temporal statistical technique uses a cylindrical window with a circular geographic base and a height defined by time interval. This technique can identify possible clusters as the center of the cylindrical window moves in space and time. The clusters represent areas that the density of events of the same type is different from outside, and have similar and consistent incidences during the clustering period^46^. Kulldorff’s spatial scan statistic is an useful tool for detecting and evaluating the spatial-temporal clusters. The spatial-temporal clustering analysis was implemented by the SaTScan software with the Bernoulli model. The significance of the spatial-temporal scan statistic was tested under the null hypothesis that dengue incidences were equally distributed in Sri Lanka from 2012 to 2016. We used the population data of each district as the control data. Maximum window size was set to conclude 50 percent of population at risk, and the window shape is circular. The number of replications was $999$. Important climate factors associated with dengue incidences were detected for different clusters by plotting monthly dengue incidence and climatic factors varying with time between January 2012 and December 2016 and comparing means and variances of meteorological data during different periods. Statistical analysis for means and variances of climate variables was performed with SPSS (version 19).

### Data availability statement

All data generated or analyzed during this study are included in this article.
